# Understanding the psychological pathways from ocean literacy to pro-environmental behavior: the mediating roles of marine responsibility and values

**DOI:** 10.3389/fpsyg.2025.1623231

**Published:** 2025-08-11

**Authors:** Linzhao Wang, Tao Gao, Yanjie Shi, Li Zhang

**Affiliations:** ^1^Mental Health Education and Counseling Center, Guangdong Ocean University, Zhanjiang, Guangdong, China; ^2^Mental Health Education and Counseling Center, Guangdong Industry Polytechnic University, Guangzhou, China; ^3^Mental Health Education Center for College Students, Kaifeng University, Kaifeng, Henan, China; ^4^College of Food Science and Technology, Guangdong Ocean University, Zhanjiang, Guangdong, China

**Keywords:** ocean literacy, pro-ocean environmental behavior, marine environmental responsibility, marine values, chain mediation model

## Abstract

**Introduction:**

This study investigates the psychological mechanisms underlying the relationship between ocean literacy and pro-environmental behavior, with a particular focus on the mediating roles of marine environmental responsibility and marine values.

**Methods:**

A large-scale survey was conducted with 1,206 university students from 23 universities across 11 provinces in China. Standardized and validated questionnaires were administered, and advanced statistical analyses, including structural equation modeling and mediation analysis, were employed to examine the hypothesized relationships.

**Results:**

Findings reveal that ocean literacy significantly enhances individuals’ sense of environmental responsibility and fosters stronger pro-environmental values. These psychological constructs, in turn, contribute to increased engagement in conservation behaviors, such as reducing plastic use and participating in beach clean-ups.

**Discussion:**

Grounded in theoretical frameworks from environmental psychology—namely the Value-Belief-Norm Theory and the Theory of Planned Behavior—this study underscores the importance of integrating cognitive, affective, and normative factors in promoting sustainable marine actions. The results offer valuable insights for the design of educational interventions and behavioral strategies aimed at advancing marine conservation efforts.

## 1 Introduction

The oceans cover nearly two-thirds of the Earth’s surface and are fundamental to global ecological stability, climate regulation, and human well-being (Masterson-Algar et al., 2022). However, intensifying human activities—including land- and sea-based pollution, overfishing, coastal habitat degradation, and the accelerating impacts of climate change —have led to unprecedented deterioration of marine ecosystems, with far-reaching consequences for biodiversity, ecosystem services, and human livelihoods (Worm et al., 2006). In response to these escalating threats, the United Nations designated 2021–2030 as the “Decade of Ocean Science for Sustainable Development,” calling for advances in ocean science and widespread behavior change through heightened public engagement (Claudet et al., 2020). For China—whose coastline exceeds 140,000 km confronting marine debris, eutrophication, and ecosystem degradation is a national priority (Chen and Martens, 2022). Technological and policy measures alone cannot ensure sustainable ocean governance. Broad public participation is essential—especially from university students, whose ocean literacy will shape the future of marine protection.

Ocean literacy has emerged as a critical construct for fostering environmental responsibility and encouraging marine conservation behaviors. Defined as an integrated understanding of the ocean’s influence on humans and humans’ influence on the ocean, ocean literacy encompasses not only scientific knowledge but also emotional attachment, ethical concern, and the capacity for informed decision-making regarding marine resources (Santoro et al., 2017; Fauville et al., 2018). The seven core principles of ocean literacy highlight the ocean’s essential roles in Earth’s systems, climate regulation, and human life, underscoring the need to develop informed and engaged citizens capable of making sustainable choices (McKinley et al., 2023). The seven core principles are as follows (Table 1). However, the psychological processes linking ocean literacy to behavior change remain underexplored.

**Table 1:** The seven essential principles of ocean literacy (Cava et al., 2005).

1. The Earth has one big ocean with many features
2. The ocean and life in the ocean shape the features of the Earth
3. The ocean is a major influence on weather and climate
4. The ocean makes Earth habitable
5. The ocean supports a great diversity of life and ecosystems
6. The ocean and humans are inextricably interconnected
7. The ocean is largely unexplored

While existing research has increasingly demonstrated that ocean literacy contributes to pro-environmental attitudes, values, and behaviors—such as reducing marine pollution, supporting sustainable consumption, and engaging in marine governance (Guest et al., 2015; Chang, 2019; Buchan et al., 2023)—growing evidence also suggests that knowledge alone is insufficient to drive behavioral change (Kollmuss and Agyeman, 2010). Prior research often treats “pro-environmental behavior” as unitary, but motivations and normative pressures differ between private actions (e.g., reducing single-use plastics) and public or collective actions (e.g., policy advocacy, beach-clean leadership). Differentiating these domains can reveal whether ocean literacy mobilizes personal lifestyle change, civic activism, or both (Husna et al., 2024). To promote sustained action, cognitive understanding must interact with deeper motivational and normative processes. In particular, constructs such as environmental responsibility and value orientations have been identified as critical mediators in this transformation. For instance, individuals who internalize a moral obligation to protect marine resources (Fransson and Gärling, 1999) are more likely to adopt both private (e.g., reducing plastic use) and public (e.g., advocacy) pro-environmental behaviors (Clayton and Myers, 2015). This underscores the importance of empirically testing integrative theoretical models that incorporate cognitive, affective, and normative factors to explain how ocean literacy is translated into meaningful behavioral outcomes.

Environmental values play an equally crucial role. Values—defined as enduring guiding principles that transcend specific contexts—shape individual attitudes, preferences, and behavioral intentions (Schwartz and Bilsky, 1987). In environmental domains, altruistic and biospheric values are consistently linked to pro-environmental attitudes and behaviors, while egoistic or hedonic values may undermine conservation efforts (Dietz et al., 2005; Steg et al., 2014). In the marine context, fostering pro-ocean environmental values can motivate individuals to engage in conservation actions, both at the individual level (e.g., sustainable consumption) and the collective level (e.g., community engagement, civic participation).

Importantly, recent research points to additional psychological factors that bridge the knowledge-action gap. For example, the self-determination theory (De Leeuw et al., 2015) suggests that autonomous motivation, grounded in intrinsic interest and personal values, is a stronger predictor of sustained pro-environmental behavior than external regulation. Moreover, the concept of connectedness to nature—the sense of emotional, cognitive, and experiential closeness to the natural world—has been identified as a key antecedent of environmental concern and behavior (McKinley et al., 2023; Nisbet et al., 2009). Environmental identity, or the degree to which individuals define themselves in relation to the natural environment, also plays a powerful motivational role, shaping intentions, moral obligations, and social norms around conservation (Clayton, 2003; O'Neill et al., 2008). These constructions underscore the need to examine not only what individuals know but also how they feel, what they value, and how they see themselves in relation to environmental challenges.

**Figure 1:**
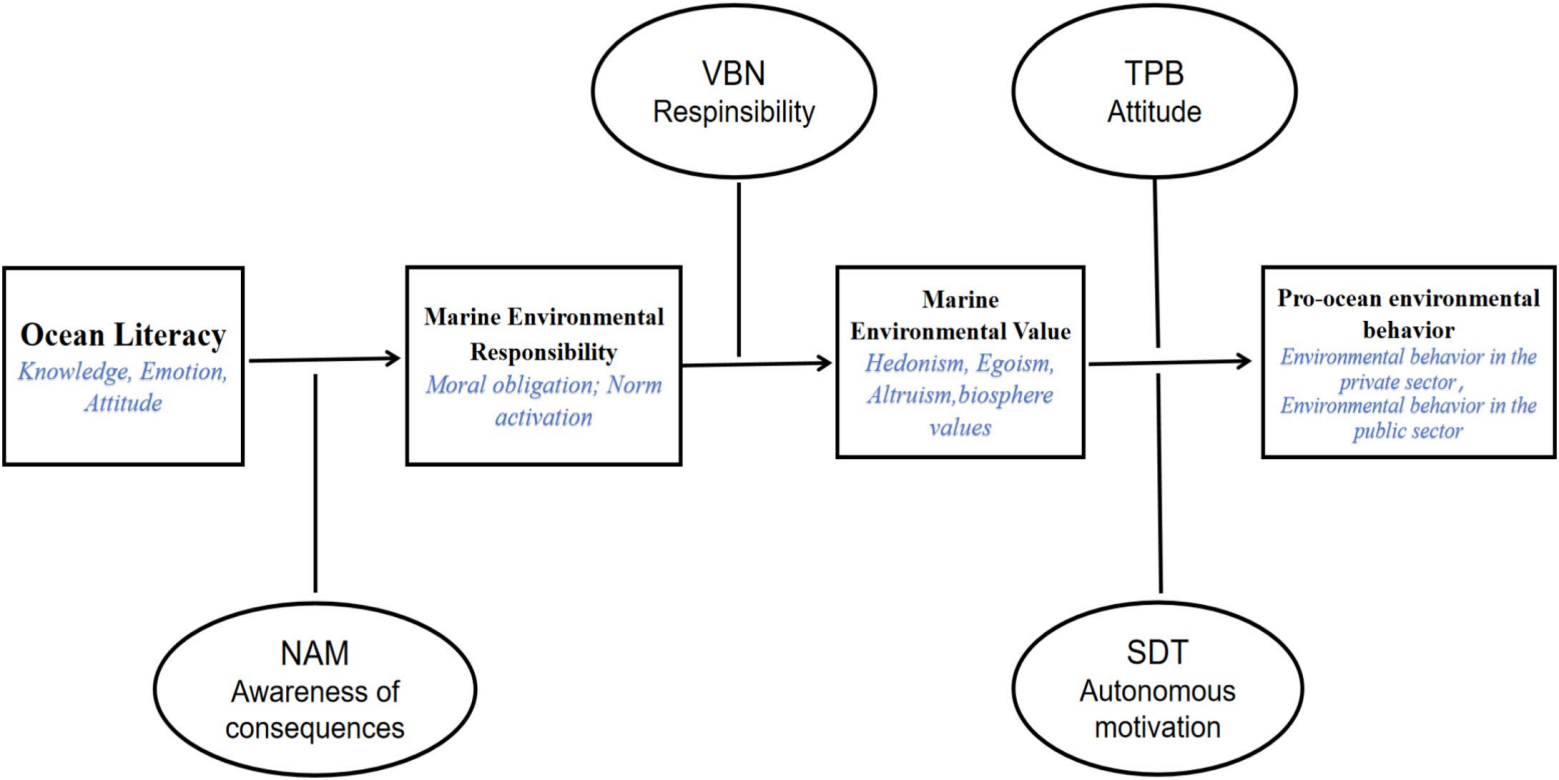
Theoretical integration and mediation model.

To synthesize these diverse psychological perspectives and clarify their interrelations, this study draws upon four well-established theoretical frameworks. To clarify the psychological mechanisms underlying pro-ocean environmental behavior, this study integrates four complementary theoretical frameworks, each offering a distinct yet interconnected perspective. The Value–Belief–Norm (VBN) theory (Stern, 2000) posits that biospheric and altruistic values shape ecological worldviews, which in turn activate personal moral norms and foster a sense of environmental responsibility—a proximal determinant of environmentally responsible behavior. Similarly, the Norm Activation Model (NAM) (Schwartz, 1977) emphasizes that awareness of environmental consequences and the ascription of personal responsibility trigger moral obligations that motivate pro-environmental action. The Theory of Planned Behavior (TPB) (Ajzen, 1991) offers a deliberative framework in which behavioral intentions are guided by individuals’ attitudes, perceived social norms, and perceived behavioral control. Complementing these perspectives, Self-Determination Theory (SDT) (De Leeuw et al., 2015) highlights the importance of autonomous motivation—rooted in internalized values—as a sustaining force for long-term environmental engagement. Drawing upon these theoretical foundations, the present study proposes an integrative chain mediation model: Ocean literacy → Marine environmental responsibility → Marine environmental values → Pro-ocean environmental behavior. As illustrated in Figure 1, the model synthesizes cognitive, normative, and motivational constructs to explain progression from knowledge and responsibility to enduring environmental action.

Despite these theoretical advances, significant gaps remain. While ocean literacy has been shown to promote environmental attitudes and behavioral intentions, few studies have empirically tested the mediating pathways through which it operates, particularly in relation to marine environmental responsibility and values. Moreover, most existing studies are concentrated in Western cultural contexts, overlooking the ways in which cultural factors shape the knowledge-attitude-behavior nexus. Research suggests that most mediation studies linking literacy to behavior have been conducted in Western, individualistic societies, where internal attitudes and self-identity dominate (Eom et al., 2016). In collectivistic China, social desirability, hierarchical norms, and communal obligations may amplify or suppress responsibility and value pathways. Understanding how ocean literacy operates within the sociocultural context of China can provide critical insights for designing culturally sensitive educational and policy interventions.

The present study seeks to address these gaps by examining the mediating roles of marine environmental responsibility and marine environmental values in the relationship between ocean literacy and pro-ocean environmental behavior among Chinese university students. By integrating cognitive, affective, motivational, and normative dimensions—grounded in TPB, VBN, NAM, and Self-Determination Theory—this study proposes a chain mediation model in which ocean literacy enhances environmental responsibility, which subsequently strengthens pro-environmental values, ultimately increasing behavioral engagement. Moreover, recognizing that cultural context plays a critical role in environmental psychology, our study focuses on China’s collectivist sociocultural setting, which may amplify the effects of normative and value-based processes. We also address potential confounds such as social desirability bias and participant academic background (e.g., marine vs. non-marine majors), which may affect self-reported behaviors.

Hypotheses.

*H1: Ocean literacy positively predicts pro-ocean environmental behavior.*

*H2: Marine environmental responsibility mediates the relationship between ocean literacy and pro-ocean environmental behavior.*

*H3: Marine environmental values mediate the relationship between ocean literacy and pro-ocean environmental behavior.*

*H4: Marine environmental responsibility and values sequentially mediate the relationship between ocean literacy and pro-ocean environmental behavior through a chain pathway.*

## 2 Materials and methods

### 2.1 Participants and procedure

A total of 1,206 undergraduate students (46.1% male, 53.9% female; aged 18–24 years, *M* = 20.39, SD = 1.45) were recruited from 23 universities across 11 provinces in China using a quota sampling approach. To ensure the sample reflected key demographic and institutional characteristics, we set quotas based on university type (general vs. marine-focused), geographic region (coastal vs. inland provinces), and academic year (first to fourth year). Of these institutions, 21 were general universities located in coastal regions, and four were marine-focused universities. Data collection was conducted via the online platform Questionnaire Star between June and July 2024. Prior to participation, informed consent was obtained from all participants. The study protocol was reviewed and approved by the Ethics Committee of Pukyong National University (Approval No. 1041386-202407-HR-106-02). Participant characteristics are presented in Table 2.

**Table 2:** Characteristics of participants (*N* = 1,206 respondents).

Category	Variable	*N*/(%)
Gender	Male	556(46.1%)
	Female	650(53.9%)
Age groups	18 and under	121(10%)
	19–22 years	1,007(83.5%)
	23 years and above	78(6.5%)
Type of college	Coastal Colleges	700(58%)
	Inland Colleges	506(42%)
Degree of relevance to marine studies	Highly Relevant	242(20.1%)
	Moderately Relevant	201(16.7%)
	Not Relevant	763(63.2%)
Professional background	Humanities	231(19.2%)
	Science	286(23.7%)
	Engineering	428(35.5%)
	Medicine	88(7.3%)
	Other	173(14.3%)
Proximity to the ocean	Coastal areas (near the sea)	806(66.8%)
	Mid-sea areas	204(17%)
	Offshore areas (far from the sea)	196(16.2%)

### 2.2 Measurement instruments

The study assessed the mediating roles of marine environmental responsibility and values in the relationship between ocean literacy and pro-ocean environmental behavior. Instruments were tested for reliability and validity. All instruments were administered in Simplified Chinese and underwent a rigorous cross-cultural adaptation process to ensure linguistic accuracy and cultural relevance. Following international guidelines (Beaton et al., 2000), the process included forward and backward translation by bilingual experts, reconciliation through committee review, and refinement for semantic and conceptual equivalence. A panel of three experts in marine education, environmental psychology, and psychometrics reviewed the translated items to ensure content validity and cultural appropriateness.

To further evaluate the psychometric properties of the adapted scales, a larger pilot study was conducted in June 2024 at Guangdong Ocean University with 168 valid responses (94.9% response rate; *M* = 19.51, SD = 1.06). The sample included students from various disciplines to ensure representativeness. All scales demonstrated strong internal consistency (overall Cronbach’s *α* = 0.954; all subscales > 0.70). Exploratory Factor Analysis (EFA) confirmed a clear factor structure with a cumulative variance explanation of 76.3%. Confirmatory Factor Analysis (CFA) on the full sample further supported the structural validity of the scales, with excellent model fit (CFI = 0.96, TLI = 0.95, RMSEA = 0.042). Based on expert judgment and factor loadings, several low-performing items were removed from the ocean literacy, environmental values, and belief scales, enhancing the reliability and conceptual clarity of the final instruments used in the main study.

#### 2.2.1 Ocean literacy

Ocean literacy was assessed using a 20-item scale adapted from Paredes Coral et al. (2022), covering three dimensions: ocean knowledge, interest, and attitudes toward marine science. Participants responded using a 5-point Likert scale ranging from 1 (strongly disagree) to 5 (strongly agree). The scale demonstrated excellent internal consistency (Cronbach’s *α* = 0.96). Confirmatory Factor Analysis (CFA) supported the construct validity of the adapted scale, with satisfactory fit indices: χ^2^/df = 2.74, CFI = 0.965, TLI = 0.951, and RMSEA = 0.048.

#### 2.2.2 Marine environmental values

Marine environmental values were assessed using a 12-item scale based on Stern and Dietz’s (1994) Value Orientation Theory and adapted by Xu (2008). The scale includes three subdimensions: egoistic, altruistic, and biospheric values. Items were rated on a 5-point Likert scale. The total scale showed excellent internal consistency (Cronbach’s *α* = 0.96), with subscale reliabilities ranging from 0.90 to 0.98. CFA supported the factorial structure, with acceptable fit indices: *χ*^2^/df = 2.83, CFI = 0.963, TLI = 0.946, RMSEA = 0.051.

#### 2.2.3 Marine environmental responsibility

Marine environmental responsibility was measured using a 4-item scale adapted from Stern et al. (2011) and Yue et al. (2020), designed to assess participants’ perceived personal and moral obligation to protect the marine environment. All items were rated on a 5-point Likert scale. The scale showed good internal consistency (Cronbach’s *α* = 0.89). CFA results indicated a good model fit: *χ*^2^/df = 2.66, CFI = 0.958, TLI = 0.947, RMSEA = 0.045.

#### 2.2.4 Pro-ocean environmental behavior

Pro-ocean environmental behavior was measured using a 15-item composite scale integrating items from Gong (2008), Zhang (2017), and Paredes Coral et al. (2022). The scale comprised two behavioral domains: private-sphere behaviors (8 items; e.g., reducing plastic use, choosing sustainable seafood) and public-sphere behaviors (7 items; e.g., participating in marine campaigns, policy advocacy). Responses were recorded on a 5-point Likert scale. The total scale demonstrated excellent reliability (Cronbach’s α = 0.96). CFA results confirmed a good model fit: *χ*^2^/df = 2.58, CFI = 0.957, TLI = 0.940, RMSEA = 0.049.

### 2.3 Data analysis

Descriptive statistics, Pearson correlation analyses, and multivariate regression analyses were conducted using IBM SPSS Statistics 26.0. To test the hypothesized chain mediation model, we used the PROCESS macro version 3.5, Model 6(Hayes, 2017). Bootstrap resampling (5,000 samples) with 95% bias-corrected confidence intervals was employed to estimate indirect effects. Gender and age were included as covariates in all analyses.

## 3 Results

### 3.1 Common method bias test

This study employed Harman’s single-factor test to assess the potential standard method bias in the research data (Zhou and Long, 2004). Principal Component Analysis (PCA) was used to extract factors without rotation to observe whether a single factor could explain most of the variance. The test results indicated 11 factors with eigenvalues greater than 1, and the most significant factor explained only 37.56% of the variance. According to Harman’s single-factor test criterion, if no single factor explains more than 40% of the variance and does not exceed half of the total explained variance, it can be considered that there is no standard severe method bias problem in the study. Therefore, the results suggest that common method bias is not a significant issue in this research, thereby enhancing the reliability and validity of the findings.

### 3.2 Descriptive statistics

Descriptive statistics and Pearson correlation analyses were conducted for ocean literacy, pro-ocean environmental behavior, marine environmental responsibility, and marine environmental values. All variables were significantly and positively correlated (*p* < 0.001), indicating the suitability of further mediation analysis. The means, standard deviations, and correlation coefficients are presented in Table 3.

**Table 3:** Correlation analysis among main variables (*N* = 1,206 respondents).

Variable	*M*	SD	Ocean literacy	Pro-environmental behavior	Marine environmental values	Marine environmental responsibility
Ocean Literacy	65.93	9.84	1			
Pro-environmental behavior	61.15	10.26	0.74***	1		
Marine environmental values	42.85	4.29	0.48***	0.55**	1	
Marine environmental responsibility	15.75	3.19	0.57***	0.79***	0.46***	1

**p < 0.01, ***p < 0.001.

### 3.3 Chain mediation effect test

#### 3.3.1 Chained mediation effect analysis

To comprehensively explore the mediating role of marine environmental responsibility and marine environmental values between ocean literacy and pro-ocean environmental behavior, a chain mediation model was established. The analysis was conducted using Hayes’s PROCESS macro Model 6 (Hayes, 2017), with 5,000 bootstrap samples and a 95% confidence interval. Gender and age were included as control variables to account for their potential confounding effects. The sample size of 1,000 participants met the recommended requirements for detecting medium-sized mediation effects with adequate statistical power (Fritz and MacKinnon, 2007).

The multiple regression model predicting marine environmental responsibility was significant [*F*(3,1202) = 200.951, *p* < 0.001], explaining 33.4% of the variance [*R*^2^ = 0.334, Adjusted *R*^2^ = 0.332]. Ocean literacy significantly predicted marine environmental responsibility (*β* = 1.223, *p* < 0.001), indicating that higher ocean literacy was associated with greater marine environmental responsibility. Gender also showed a small but significant effect (*β* = 0.105, *p* = 0.005), suggesting that female students reported slightly higher responsibility scores. Age was not a significant predictor (*β* = −0.001, *p* = 0.835). The regression analysis predicting marine environmental values was significant as well [*F*(4,1201) = 120.321, *p* < 0.001], explaining 28.6% of the variance (*R*^2^ = 0.286, Adjusted *R*^2^ = 0.284). This supports the theoretical assumption that responsibility fosters value development. Ocean literacy had a significant positive effect on marine environmental values (*β* = 0.318, *p* < 0.001), suggesting that increased ocean literacy strengthens marine environmental values. Additionally, marine environmental responsibility positively predicted marine environmental values (*β* = 0.116, *p* < 0.001), confirming its mediating role between ocean literacy and marine environmental values. The final regression model predicting pro-ocean environmental behavior was highly significant [*F*(5,1200) = 775.652, *p* < 0.001], explaining 76.4% of the variance (*R*^2^ = 0.764, Adjusted *R*^2^ = 0.763). Ocean literacy significantly and directly affected pro-ocean environmental behavior (*β* = 0.389, *B* = 0.702, *p* < 0.001). Marine environmental responsibility and marine environmental values served as significant mediators, enhancing the positive impact of ocean literacy on pro-ocean environmental behavior, with standardized coefficients of *β* = 0.509 (*p* < 0.001) and *β* = 0.128 (*p* < 0.001), respectively (Table 4). To better understand the context, we conducted a subgroup descriptive analysis. Among the 1,206 participants, 58% (*n* = 700) were enrolled in coastal universities, and 20.1% (*n* = 242) were from marine-focused programs. This may help explain the strong correlation between ocean literacy and pro-environmental behavior, even though the mean ocean literacy score was moderate (*M* = 65.93, SD = 9.84). Participants’ academic and institutional environments likely contributed to the behavioral salience and application of ocean-related knowledge.

**Table 4:** Regression analysis between the main variables (*N* = 1,206 respondents).

Outcome variables	Predictor variables	*R*	*R* ^2^	*F*	*β*	*t*
Marine environmental responsibility		0.58	0.33	200.95***	0.70	3.94***
	Ocean literacy				1.22	24.54***
Marine environmental value		0.54	0.29	120.32***	2.22	26.93***
	Ocean literacy				0.32	11.22***
	Marine environmental responsibility				0.12	8.70***
Pro-environmental behavior		0.87	0.76	775.62***	−0.48	−4.12***
	Ocean literacy				0.70	21.42***
	Marine environmental responsibility				0.44	28.71***
	Marine environmental value				0.25	7.73***

***p < 0.001.

These results collectively suggest that marine environmental responsibility and values play critical mediating roles linking ocean literacy to pro-ocean environmental behavior. Enhancing ocean literacy among university students can effectively foster stronger marine environmental responsibility and reinforce their environmental values, thereby promoting active pro-environmental behaviors.

#### 3.3.2 Testing of multiple chained mediating effects

The mediation analysis further clarified how ocean literacy influences pro-ocean environmental behavior. The total indirect effect was significant (effect = 0.646, BootSE = 0.040, 95% CI [0.569, 0.729]), indicating robust mediation. Three significant indirect pathways emerged: Path 1 (Ocean literacy → Marine environmental responsibility → Behavior): This was the dominant mediation path (effect = 0.533, BootSE = 0.036, 95% CI [0.465, 0.604]), highlighting the pivotal role of responsibility; Path 2 (Ocean literacy → Marine environmental values → Behavior): A smaller but significant indirect effect was observed (effect = 0.078, BootSE = 0.014, 95% CI [0.053, 0.108]), confirming the role of environmental values; Path 3 (Ocean literacy → Responsibility → Values → Behavior): The sequential chain mediation was also supported (effect = 0.035, BootSE = 0.006, 95% CI [0.023, 0.048]), indicating that ocean literacy enhances behavior partly through a value-driven pathway initiated by responsibility.

**Figure 2:**
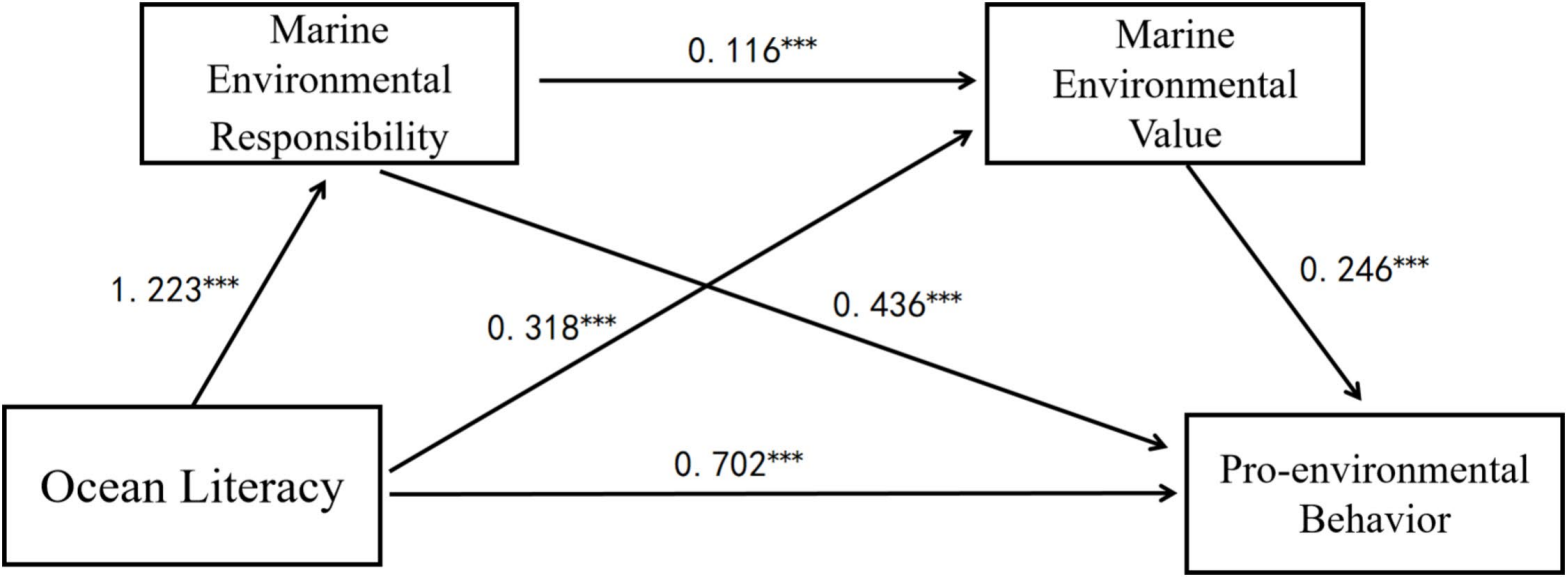
Results of a serial mediation model. ***p < 0.001.

These findings validate the proposed chain mediation model and reinforce the centrality of marine environmental responsibility in shaping pro-environmental behavior. Notably, although the mean score of ocean literacy was close to the theoretical average, its high correlation with behavioral outcomes may reflect the synergistic role of affective and normative factors, particularly in students with relevant educational exposure. Gender differences in responsibility, while modest, align with previous studies suggesting higher environmental concern and moral engagement among females. These results offer practical implications for educational interventions aiming to translate ocean literacy into meaningful behavioral change (see Table 5 and Figure 2).

**Table 5:** Mediation pathway analysis.

Mediation pathway	Effect size	Boot SE	BootLLCI	BootULCI
Ocean literacy → Marine environmental responsibility →Pro-environmental behavior	0.53	0.04	0.47	0.60
Ocean literacy → Marine environmental value →Pro-environmental behavior	0.08	0.01	0.05	0.11
Ocean literacy → Marine environmental responsibility → Marine environmental → value → Pro-environmental behavior	0.04	0.01	0.02	0.05
Total effect	0.65	0.04	0.57	0.73

“Boot SE,” “Boot LLCI,” and “Boot ULCI” respectively denote the standard error of the indirect effects estimated via the bias-corrected percentile method, and the lower and upper bounds of the 95% confidence interval.

## 4 Discussion

### 4.1 The direct influence of ocean literacy on pro-ocean environmental behavior

The research findings indicate that enhancing individuals’ ocean literacy can strengthen their pro-ocean environmental behavior. This aligns with the study by Paredes Coral et al. (2022), which emphasizes the central role of education in raising awareness of environmentally responsible behaviors. However, it is noteworthy that while the mean level of ocean literacy was only moderate across the sample (*M* = 65.93, SD = 9.84), it still showed the strongest correlation with pro-environmental behavior (*r* = 0.74, *p* < 0.001). One plausible explanation is that even modest gains in marine knowledge and awareness may activate underlying psychological constructs such as personal responsibility and emotional concern, thereby disproportionately enhancing behavioral intentions. This is particularly relevant in samples where students—although not all enrolled in marine-related majors—may have strong contextual exposure to ocean issues due to geographic or educational proximity. Although type of college and degree relevance to marine studies were not significant predictors in preliminary models, we acknowledge this as a limitation and encourage future models to incorporate professional background as potential moderators. Brennan et al. (2019) also point out that high levels of ocean literacy can directly promote individuals to take active marine conservation measures, such as reducing pollution, supporting sustainable ocean policies, and participating in marine protection activities. Enhancing marine knowledge and awareness through education increases individuals’ recognition of the importance of marine conservation and stimulates their willingness to take concrete protective actions (Vesely et al., 2020; McKinley et al., 2023). From a psychological perspective, this suggests that knowledge about marine systems enhances not only cognitive awareness but also motivational and attitudinal readiness for action. Prior studies in environmental psychology emphasize that knowledge is a foundational but insufficient condition for behavior change (Klöckner, 2013; McKinley and Burdon, 2020). The cognitive-affective model suggests that cognitive factors such as environmental literacy interact with emotional engagement and behavioral intentions to shape actual behavior. In line with this, our findings highlight that marine education programs should not merely transfer knowledge but also foster agency, self-efficacy, and behavioral control, which are known predictors of pro-environmental action (Ajzen, 1991).

Moreover, increased marine knowledge helps individuals better understand the complexity of marine ecosystems and their significance for global environmental health. This cognition enhances individuals’ sense of responsibility, driving them to take action to protect this fragile ecosystem (Klöckner, 2013). Ocean literacy also encompasses attitudes and values toward marine environmental issues and critical psychological factors driving pro-ocean environmental behavior (McKinley et al., 2023). Students in coastal areas, who are more frequently exposed to marine environments, may develop stronger place attachment and emotional connectedness to nature—factors identified as robust predictors of pro-environmental intentions and behaviors (Manning and Clayton, 2018). Furthermore, our data suggest that ocean literacy more strongly predicts private-sphere behaviors (e.g., plastic use reduction) than public-sphere actions (e.g., environmental advocacy), highlighting the need for differentiated educational strategies tailored to behavior domains (Husna et al., 2024). Thus, interventions that leverage local environmental contexts and provide experiential learning opportunities may more effectively convert ocean literacy into sustained behavioral engagement.

This study underscores the central role of education in shaping environmental behavior, especially when the geographical environment allows students to engage with natural resources directly. For example, college students in coastal areas have their environmental awareness and sense of urgency strengthened through daily interactions with the ocean. This direct experience not only provides learning opportunities but also becomes a powerful motivator for action. However, we also observe that merely increasing marine knowledge does not fully translate into action; emotional engagement and the stimulation of behavioral intentions are also needed (Lee, 2022). Therefore, in designing marine education and advocacy strategies, emphasis should be placed on cultivating emotional connections and behavioral skills to ensure that knowledge can be transformed into actual protective actions.

In summary, the direct relationship between ocean literacy and pro-ocean environmental behavior highlights the power of education, achieved not only by providing information but also by shaping attitudes and stimulating behavior. By integrating education, social influence, and policy tools, public awareness and participation in marine conservation can be effectively enhanced. This provides important insights for policymakers and educators, suggesting that educational content should incorporate practical activities and emotional education to promote sustainable marine conservation actions.

### 4.2 The mediating role of marine environmental responsibility and marine environmental values

This study reveals how ocean literacy promotes pro-ocean environmental behavior by enhancing marine environmental responsibility and values. Specifically, ocean literacy significantly improves individuals’ sense of marine environmental responsibility, positively influencing marine environmental values and promoting pro-ocean environmental behavior. This chain mediation relationship emphasizes the critical bridging role of marine environmental responsibility between ocean literacy and pro-ocean environmental behavior. This pathway is theoretically grounded in the Value-Belief-Norm (VBN) theory (Stern, 2000), which posits that values influence behavior through the activation of personal norms such as environmental responsibility. Our findings affirm this mechanism within the marine domain, especially among Chinese university students. Our findings are consistent with those of Hines et al. (1987), who emphasized the significant role of environmental responsibility in predicting pro-environmental behavior. This study elucidates the chained mediating role of ocean literacy in promoting pro-ocean environmental behaviors by enhancing marine environmental responsibility and values. This mediating effect aligns closely with the classic Value-Belief-Norm (VBN) theory (Stern, 2000) and also extends the applicability of the VBN theory within the context of marine environmental conservation, providing a solid empirical basis. Specifically, an increase in individual responsibility toward the marine environment can further strengthen environmental values, ultimately driving concrete conservation actions. This is consistent with recent studies by Janmaimool and Chudech (2020) and Yue et al. (2020), who noted that enhancing a sense of individual responsibility is particularly crucial among young groups and is a key driver for advancing societal goals toward sustainable development. The Norm Activation Model (NAM) complements this interpretation by illustrating how awareness of environmental consequences can activate moral obligations and responsibilities, which, in turn, stimulate value-aligned actions (Schwartz, 1977). Our data provide empirical support for NAM in the marine conservation context.

Through empirical data, this study reinforces the findings of Liobikienė and Juknys (2016), namely that environmental values significantly affect individuals’ pro-environmental behavior, especially in marine conservation. Enhancing marine environmental values has been proven to be an essential psychological mechanism for promoting pro-ocean environmental behavior, including a focus on altruism and ecocentrism. When individuals’ ocean literacy improves, they are more inclined to recognize the long-term impact of their actions on the environment, thereby taking more responsible actions to support marine conservation. This study further extends the findings of Kaiser et al. (1999) by demonstrating the critical role of values in promoting pro-environmental behaviors, especially in contexts where pro-ocean actions are directly linked to individual self-interest. Therefore, educational policies should place greater emphasis on how to effectively balance self-interested and altruistic biospheric values, to inspire more individuals to actively participate in conservation efforts.

These results reinforce the role of moral emotions, such as guilt, pride, and moral outrage, in translating environmental concern into action (Schwartz and Bilsky, 1987; Janmaimool and Chudech, 2020). As Dunlap and Van Liere (1978) elaborated, ocean literacy includes understanding the basic knowledge and values of the marine environment, which is crucial in motivating the public to participate actively in marine conservation behaviors. Interestingly, we also observed significant gender differences in marine environmental responsibility, with female participants scoring higher than males. This may reflect gendered differences in empathy and moral socialization, as reported in prior studies (Zelezny et al., 2000). Future work should investigate how gender norms intersect with environmental responsibility and behavior. Educational interventions should therefore focus not only on cognitive development but also on the emotional and normative domains, helping individuals internalize environmental norms and feel personally responsible for conservation efforts.

### 4.3 Empirical validation of the chain mediation model

The empirical results of this study reveal a significant psychological mechanism through which ocean literacy influences pro-ocean environmental behavior among university students, highlighting the mediating roles of marine environmental responsibility and marine environmental values. Specifically, the findings indicate a positive chain mediating effect, suggesting that higher levels of ocean literacy effectively enhance individuals’ sense of marine environmental responsibility, which subsequently promotes the development of stronger marine environmental values, ultimately leading to increased pro-ocean environmental behaviors.

These results align with and expand upon key theoretical frameworks, particularly the Value-Belief-Norm (VBN) model (Stern, 2000) and the Theory of Planned Behavior (TPB) (Ajzen, 1991). The VBN model emphasizes how personal values and beliefs activate a sense of moral obligation, which in turn drives pro-environmental behavior. In contrast, the TPB highlights the role of perceived behavioral control, attitudes, and subjective norms in shaping intentional actions. Our findings suggest that enhancing marine literacy activates both normative commitments and volitional mechanisms. Specifically, marine environmental responsibility emerges as a critical psychological mediator that translates general environmental beliefs into concrete values and behaviors—supporting the core premise of the VBN framework. At the same time, consistent with TPB, our results indicate that knowledge alone is insufficient to change behavior; instead, ocean literacy must interact with attitudinal and control-related factors to generate behavioral intentions (Kollmuss and Agyeman, 2010; Qiu et al., 2022). In this sense, marine environmental responsibility and values function similarly to TPB constructs, facilitating the translation of knowledge into sustained pro-ocean actions.

The chain mediation model identified in this study reveals a more refined psychological pathway than previously suggested in the direct-correlation framework proposed by Corral-Verdugo and Armendariz (2000). While their model emphasized a linear association from environmental beliefs to behavior, our findings indicate that environmental responsibility and value formation act as sequential mediators. This clarifies how abstract cognitive constructs—such as ocean literacy—are translated into concrete conservation actions via intermediate psychological processes.

These results align with emerging evidence that emotional and relational factors are critical in driving pro-environmental behavior. Prior studies (Manning and Clayton, 2018; Liobikienė and Juknys, 2016) have shown that affective bonds with nature strongly predict ecological action. Similarly, our findings indicate that higher ocean literacy is associated with heightened feelings of responsibility and stronger biospheric and altruistic value orientations. These results support the view that emotional engagement is a necessary component of effective marine-conservation education and policy. Equally important, this study affirms the pivotal role of well-designed educational interventions. Effective programs must go beyond delivering factual knowledge to engage learners’ moral reasoning and cultivate environmental values—key psychological drivers of lasting behavioral change. This reinforces the growing consensus that transformative learning is essential for fostering deep and sustained ocean stewardship (McPherson et al., 2018).

These insights have direct implications for intervention design. Because our sample comprised Chinese university students, cultural values such as collectivism, social harmony, and educational meritocracy may amplify the influence of subjective norms and collective efficacy on pro-ocean action. In collectivist contexts, ocean literacy tends to be more strongly associated with low-risk, socially encouraged private-sphere behaviors, while public-sphere actions often depend on perceived institutional efficacy and trust (Eom et al., 2016). Therefore, interventions combining scientific content with emotionally engaging narratives or immersive experiences (e.g., virtual-reality ocean explorations) could enhance moral motivation. At the same time, programs that promote collective engagement and strengthen institutional credibility may help shift action beyond the private sphere. Nevertheless, the generalizability of these mechanisms requires cautious interpretation, as they may function differently in individualistic societies that prioritize personal autonomy over social norms.

Future research should further examine how academic background, college type, and the relevance of students’ majors to marine studies influence the strength and structure of the mediating pathways identified in this study. Although these variables were collected, they were not included in the mediation model—an omission that future studies should address to enhance explanatory depth. Additionally, the observed gender difference in environmental responsibility warrants further investigation into the underlying psychological or sociocultural mechanisms. To improve intervention design, future studies could compare the effects of cognitive (knowledge-based) versus emotional (value- and identity-based) components of marine education, identifying which more effectively mobilize behavioral change across diverse populations. Given the cultural specificity of this study, cross-cultural replication is also necessary to test the generalizability of these mechanisms and to identify how cultural norms and values moderate the effects of ocean literacy on pro-environmental behavior. Such insights will be critical for developing culturally adaptive, evidence-based strategies in global marine education.

## 5 Conclusion

This study demonstrates that ocean literacy plays a central role in fostering pro-ocean environmental behavior, primarily through the mediating effects of marine environmental responsibility and, secondarily, through marine environmental values. By empirically validating a chain mediation model, the research clarifies how cognitive understanding can evolve into sustained conservation actions via affective and normative pathways.

First, ocean literacy significantly enhances individuals’ sense of moral responsibility toward the marine environment, which serves as the most potent psychological driver of pro-environmental behavior. This effect is further reinforced by the development of altruistic and biospheric values, which strengthen individuals’ internal motivation to act.

Second, the findings affirm that knowledge alone is insufficient. Educational interventions must target deeper psychological dimensions—such as moral obligation, emotional engagement, and identity-based values—to activate long-term behavioral change. Programs that incorporate both informational and affective components are more likely to produce meaningful conservation outcomes.

Third, this study underscores the practical implications of targeting specific psychological pathways in marine education. Programs that cultivate a sense of environmental responsibility and strengthen biospheric and altruistic values—rather than relying solely on factual instruction—are more likely to motivate sustained conservation behaviors. In China’s collectivist cultural context, where normative alignment and group-based values are emphasized, embedding emotional and moral components into educational initiatives may further enhance their effectiveness and reach.

Together, these results contribute to environmental psychology by unpacking the layered psychological mechanisms that connect literacy with action in a marine context. Future research should explore how cultural, demographic, and contextual variables—such as educational background or societal norms—moderate these relationships. Cross-cultural validation and longitudinal designs are particularly needed to inform the development of culturally sensitive, evidence-based marine education strategies that can drive global ocean sustainability.

## Data Availability

The original contributions presented in the study are included in the article/supplementary material, further inquiries can be directed to the corresponding author.
